# Cigarette Smoking Modulation of Saliva Microbial Composition and Cytokine Levels

**DOI:** 10.3390/ijerph15112479

**Published:** 2018-11-07

**Authors:** Mary Rodríguez-Rabassa, Pablo López, Ronald E. Rodríguez-Santiago, Antonio Cases, Marcos Felici, Raphael Sánchez, Yasuhiro Yamamura, Vanessa Rivera-Amill

**Affiliations:** 1 AIDS Research Infrastructure Program, Ponce Research Institute, Ponce Health Sciences University, 395 Dr. Luis F. Sala Street, Ponce, PR 00716-2348, USA; marodriguez@psm.edu (M.R.-R.); plopez@psm.edu (P.L.); erodriguez15@stu.psm.edu (R.E.R.-S.); rsanchez@psm.edu (R.S.); bonyamam@gmail.com (Y.Y.); 2 Clinical Psychology Program, School of Behavioral & Brain Science, Ponce Health Sciences University, Ponce, PR 00716-2348, USA; 3 Tobacco Control and Oral Health Division, Department of Health, Commonwealth of Puerto Rico, San Juan, PR 00716-2348, USA; acases@salud.gov.pr (A.C.); marcos.felici@upr.edu (M.F.)

**Keywords:** tobacco, smoking, saliva, microbiome, cytokines

## Abstract

Tobacco use has been implicated as an immunomodulator in the oral cavity and contributes to the development of oral cancer. In the present study, we investigated the effects of cigarette smoking on bacterial diversity and host responses compared to healthy nonsmoking controls. Saliva samples were collected from eighteen smokers and sixteen nonsmoking individuals by passive drool. The 16S rRNA gene was used to characterize the salivary microbiome by using the Illumina MiSeq platform. Cytokine and chemokine expression analyses were performed to evaluate the host response. Significant differences in cytokine and chemokine expression levels of MDC, IL-10, IL-5, IL-2, IL-4, IL-7, adrenocorticotropic hormone (ACTH), insulin, and leptin were observed between smokers and nonsmokers. Taxonomic analyses revealed differences between the two groups, and some bacterial genera associated with the smokers group had correlations with hormones and cytokines identified as statistically different between smokers and nonsmokers. These factors have been associated with inflammation and carcinogenesis in the oral cavity. The data obtained may aid in the identification of the interactions between the salivary microbiome, host inflammatory responses, and metabolism in smokers.

## 1. Introduction

Although policies to control cigarette smoking in the population have been successfully implemented in Puerto Rico, it is still an important public health problem on the island [1]. In 2016, the prevalence of adults who smoked in Puerto Rico was 10.6% [2]. Tobacco use is a key modifiable behavioral risk factor associated with chronic diseases [3]. Cigarette smoke has been linked to many common medical conditions such as cancer, coronary heart disease, chronic obstructive pulmonary diseases, and periodontitis [4,5,6,7]. Recent studies have established that cigarette smoking has a significant effect on the microbiota and cytokines expression of the buccal mucosa [8,9,10,11,12]. These factors have been associated with inflammation and carcinogenesis in the oral cavity and other body compartments [13,14]. The incidence rate of oral cancer in Puerto Rico is high and may be related in part to tobacco use [15,16]. Interestingly, men are still more likely to smoke (32.1%) than women (25.1%) [17], which coincides with a higher incidence of oral cavity and pharyngeal carcinomas in men in the Puerto Rican population [18]. Oral cancer is the eighth most prevalent cancer worldwide and constitutes a significant global public health problem [19,20]. Oral cancers have multifactorial origins such as smoking cigarettes, heavy alcohol consumption, bacterial translocation, and human papilloma virus infection (HPV), among others [21,22,23].

Several recent studies have suggested that environmental factors (e.g., diet and smoking) and host susceptibility (e.g., stress, anxiety, and depression) affect the equilibrium of the oral microbiome (dysbiosis) and expand the growth of oral pathogens that may have effects on other parts of the body [24,25,26]. For example, cancer in digestive organs (colorectal and pancreatic) has been associated with oral microflora (*Porphyromonas gingivalis*, *Fusobacterium nucleatum*), and in many cases with smoking [27,28,29,30,31]. In addition, microbial composition influences the development and function of innate and adaptive branches of the immune system [32].

The principal objective of the study was to analyze the relationship between cigarette smoking, salivary microbiome composition, and host response among adult Puerto Rican smokers by using high-throughput 16S rRNA gene sequencing and Luminex xMAP technology. This information may aid in the identification of the interactions between the salivary microbiome, host immune responses, and metabolic processes in smokers.

## 2. Methods

The current study was conducted following the Declaration of Helsinki, and the protocol was approved by the Institutional Review Board of the Ponce Medical School Foundation, Inc. (IRB approval no: 150209-YY). All participants signed their informed consent before sample collection and completion of study questionnaires.

### 2.1. Study Subjects

This cross-sectional study involved the collection of participants’ saliva and depression symptomatology as described below. Thirty-four subjects were recruited for this study, 16 nonsmokers (sex: 6 males, 10 females) and 18 current smokers (sex: 10 males, 8 females). Fifty percent of the current smokers were recruited from the Puerto Rico Department of Health Quitline (*n* = 9) and 50% from the Puerto Rican general population (*n* = 9). All nonsmokers were recruited from the Puerto Rican general population.

### 2.2. Depression Symptomatology

The Patient Heath Questionnaire-9 (PHQ-9) was used to assess depression symptoms. This 9-item self-report scale evaluates how often the described symptom bothered the participant over the 2 weeks prior to the test being filled out. Each of the items can be scored from 0 (not at all) to 3 (nearly every day). The general score can range from 0 to 27 and is interpreted as follows: a score of 0–4 means that the subject has minimal or no symptoms, 5–9 signifies mild depression, 10–14 denotes moderate depression, 15–19 suggests moderately severe depression, and 20–27 signifies that the subject has severe depression. Its validity and reliability as a diagnostic measure, as well as its utility in assessing depression severity and monitoring treatment response, are well-established [33,34,35,36,37].

### 2.3. Sample Collection, DNA Extraction, and Amplification

Approximately 1 mL of saliva was collected by passive drool into an UltraSal-2 saliva collection device (Oasis diagnostics, Vancouver, WA, USA). The samples were aliquoted and stored at −20 °C until processing. Bacterial DNA was extracted from saliva using the QIAamp DNA Microbiome Kit (Qiagen, Hilden, Germany) according to the manufacturer’s recommendations. The conserved 16S V3-V4 region was amplified using PCR protocol. We performed the 16S rRNA gene amplification by using FastStart master mix from Roche (Roche Diagnostics, Mannhein, Germany). The forward and reverse primers used for the amplification were 16F (5′-TCGTCGGCAGCGTCAGATGTGTATAAGAGACAGCCTACGGGNGGCWGCAG-3′) and 16R (5′-GTCTCGGTGGGCTCGGAGATGTGTATAAGAGACAGGACTACHVHHHTATCTAATCC-3′), respectively [38]. The PCR conditions used were as follows: A denaturing step at 95 °C for 3 min, followed by 25 cycles at 95 °C for 30 s, 55 °C for 30 s, and 78 °C for 30 s, and a final extension at 72 °C for 5 min. In order to detect possible contamination during the procedures, the amplifications were carried out with an appropriate negative control. Each amplicon was confirmed by 1.5% agarose gel.

### 2.4. Illumina MiSeq Sequence Determination by Nextera XT

After PCR amplification, we used a Qubit dsDNA HS assay kit and a Qubit fluorometer (Life Technologies, Carlsbad, CA, USA) to quantify the concentration of the amplicons. The Nextera XT DNA Sample Preparation Kit (Illumina, Sand Diego, CA, USA) was used according to the manufacturer’s instructions to generate paired-end DNA libraries. Briefly, the PCR products were fragmented and tagged with an adapter, and the library was purified by using AMPure XP beads (Beckman Coulter, Indianapolis, IN, USA). A normalization procedure was performed to ensure equal library representation during sequencing. Each sample was pooled into a 1.5 mL tube and heated at 96 °C for 2 min. The denatured library was placed on ice for 5 min; PhiX (12.5 pM) library control was added to provide quality control checks. The amplicon library was loaded onto the Illumina MiSeq flow cell. A sequencing run of 2 × 250 bp MiSeq paired-end reads was performed. The data obtained were base-called, and reads with the same barcode were collected and assigned to a sample on the instrument. After the procedure, the software generated analysis output in the FASTQ file format.

### 2.5. Metagenomics Data Quality Control and Analysis

MiSeq data was extracted, decompressed, and analyzed using the QIIME software (v.1.9.0) (University of Colorado, Boulder, CO, USA) [39]. Forward and reverse reads (FASTQ file) of each sample were joined using the *join_paired_ends.py* script with QIIME default parameters, and the quality of sequences was assessed using FastQC software (v.10.1, Babraham Institute, Cambridge, UK) (www.bioinformatics.babraham.ac.uk/projects/fastqc/). The resulting file containing all sequences was length and quality trimmed by discarding sequences shorter than 75 nucleotides and a quality threshold of 20 using Trim Galore! software (Babraham Institute, Cambridge, UK) (htps://www.bioinformatics.babraham.ac.uk/projects/trim_galore/). The resulting files were converted to FASTA using the *fastq_to_fasta* script (FASTX-Toolkit, Hannon Lab, Cold Spring Harbor, NY, USA, http://hannonlab.cshl.edu/fastx_toolkit/license.html) and then used to determine the taxonomy composition of each sample (*pick_closed_reference_otu.py* script) with a 97% similarity threshold using the Greengenes reference dataset (gg_otus_13_8-release). The beta diversity analyses were produced with the core_diversity_analyses.py from QIIME [40]. The rarefaction of the Operational Taxonomic Unit (OTU) table for alpha diversity was 4394. We also calculated beta diversity with unweighted UniFrac, as well as microbe relative abundance files [41,42]. The OTU table was rarefied to the same number of OTUs using the *single_rarefication.py* QIIME command. The rarefied OTU table was used as input for Explicet (v.2.10.5, University of Colorado, Boulder, CO, USA) to create stacked bar charts and other statistical data [43].

The sequence data were submitted to NCBI BioProject (http://www.ncbi.nlm.nih.gov/bioproject) under accession number PRJNA407051.

### 2.6. Cytokine Assay

Proinflammatory and anti-inflammatory cytokine/chemokine concentrations in saliva were calculated by using a Milliplex Human Cytokine/Chemokine Magnetics Bead Panel (TNF-α, IL-12 (p70), MDC, IL-10, IFN-γ, TNF-β, IL-1β, IL-5, IL-2, IL-6, IL-4, IL-1RA, IL-13, IL-17, IL-7, and GM-CSF) and a Human Bone Magnetic Bead Panel (insulin, leptin, and adrenocorticotropic hormone (ACTH)). The procedures were performed according to the manufacturer’s recommendations (Millipore, Billerica, MA, USA). A Luminex MAGPIX instrument with xPonent software version 4.2 (Luminex, TX, USA) and a Milliplex Analyst version 3.5.5.0 (VigeneTech, Carlisle, MA, USA) were used to collect the data and for analyses, respectively. Briefly, a minimum of 50 microsphere events were acquired from each analyte, and cytokine/chemokine concentrations were calculated by measuring the median fluorescent intensity (MFI) in a 5-parameter curve-fitting method [44]. Measurements were performed in duplicate.

### 2.7. Microbial Biomarkers and Correlation Model

To determine smoker microbial biomarkers, we used LEfSe [45] to compare the bacterial taxonomic composition of the smoking and nonsmoking groups. The taxonomy was evaluated at the genus level, and it was given a *p*-value of 0.05 to be considered significant and a minimum threshold of 3.0 in the linear discriminant analysis (LDA). To understand correlations between the bacteria, cytokines, and hormones, we generated a correlation network. The correlations and the network were generated using qgraph in R 3.4.0 (University of Amsterdam, Amsterdam, The Netherlands) [46].

### 2.8. Statistical Analysis

The statistical significance between nonsmoker and smoker variables (depression and cytokine expression) were performed using a nonparametric Mann-Whitney-Wilcoxon test implemented in SPSS version 20 (SPSS Inc., Chicago, IL, USA). The level of statistical significance was set at *p* < 0.05. The tests of significance of unweighted UniFrac distance were performed using a two-sided Student’s two-sample *t*-test. The nonparametric *p*-values (Bonferroni-corrected) were calculated using 999 Monte Carlo permutations implemented by QIIME (University of Colorado, Boulder, CO, USA) [39]. A Spearman correlation test was used for the correlation network.

## 3. Results

### 3.1. Demographic Parameters of Study Participants

A total of 34 participants were enrolled in the study, including 16 nonsmokers and 18 current smokers. None of the participants were taking antibiotics. The participants from the smoker group mostly had over three years of smoking history (83.3%). Half of the participants reported light/moderate cigarette consumption (<20 cigarettes per day), whereas the others reported being heavy cigarette users (≥20 cigarettes per day). Demographic data showed that the individuals in the smoker group were predominantly male (55.6%), whereas the nonsmoker group was mostly female (62.5%). The mean age for individuals in the smoker group was 45 years (range 33–49), whereas in the nonsmoker group it was 34 years (range 28–50). The majority of the participants in the smoker group (67.0%), as well as in the nonsmoker group (75.0%), had a college education. The participants from the nonsmoker group showed higher alcohol use (68.8%) than those from the smoker group (38.9%). No statistically significant differences were detected for age, sex, education level, and alcohol use (Table 1). However, the PHQ-9 mean scores showed significantly increased (*p* = 0.02) depression symptomatology in smokers when compared to nonsmokers (Table 1).

### 3.2. Microbial Sequencing and Bacterial Diversity

**Figure 1:**
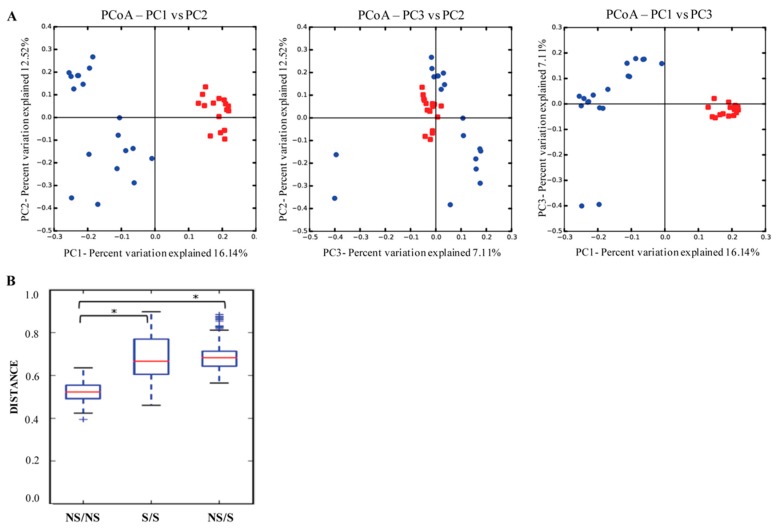
Clustering of samples from nonsmokers and smokers. (**A**) for beta diversity, principal coordinate analysis plots (PCoA) based on unweighted UniFrac distance were used to visualize the variation present in all samples by smoking status. Emperor web browser was used to perform the analysis. Significant cluster was observed in the saliva microbial community among smokers (blue dots) and nonsmokers (red dots). (**B**) unweighted UniFrac plot representing the intraindividual variability within nonsmokers (NS) and smokers (S). The asterisks (*) indicate a significant difference (nonparametric *p*-value with Bonferroni correction), where *p* = 0.01.

**Figure 2:**
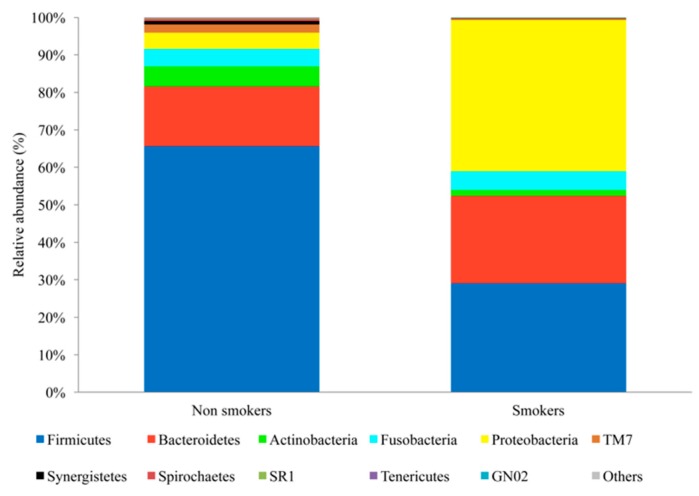
Taxonomic composition of nonsmokers and smokers at the phylum level. FASTA files were used to determine the taxonomy composition of each sample with a 97% similarity threshold using the Greengenes reference dataset.

According to the beta diversity of the unweighted UniFrac analysis, the smoker and nonsmoker groups were significantly different (*p*-value < 0.05). The salivary microbial population between the two groups differed by the most abundant bacteria (Figure 1A). In addition, the intraindividual variability was greater in smokers than in nonsmokers (Figure 1B). The five phyla that were more abundant in nonsmokers’ saliva samples were Firmicutes (66%), Bacteroidetes (16%), Actinobacteria (5%), Fusobacteria (5%), and Proteobacteria (4%), which was 96% of all sequences. In contrast, Proteobacteria (40%), Firmicutes (29%), Bacteroidetes (23%), Fusobacteria (5%), and Actinobacteria (2%) dominated in smoker samples and represented 99% of all sequences (Figure 2).

**Figure 3:**
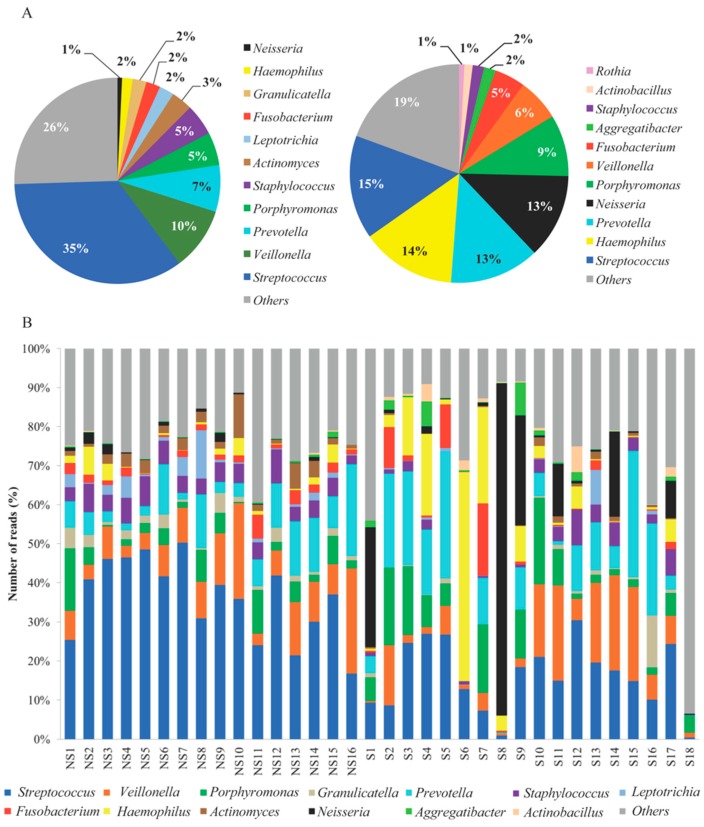
Composition of the major genera observed in nonsmoker (NS; *n* = 16) and smoker (S; *n* = 18) saliva samples. (**A**) comparison of the taxonomic analyses at the genus level by group. (**B**) composition of the major genera by study participant. The bacterial abundance was established by the analysis of the 16S rRNA gene using a Quantitative Insights into Microbial Ecology (QIIME) pipeline.

At the genus level, *Streptococcus* was most prevalent in both groups, although at a lower percentage in the smoker group (Figure 3). The bacterial compositions (the top ten) in the nonsmoker group samples were as follows: *Streptococcus* (35%), *Veillonella* (10%), *Prevotella* (8%), *Porphyromonas* (5%), *Staphylococcus* (5%), *Actinomyces* (3%), *Leptotrichia* (2%), *Fusobacterium* (2%), *Granulicatella* (2%), and *Haemophilus* (2%). The bacterial compositions in the smoker group samples differed from the nonsmoker group: *Streptococcus* (15%), *Haemophilus* (14%), *Prevotella* (13%), *Neisseria* (13%), *Porphyromonas* (9%), *Veillonella* (6%), *Fusobacterium* (5%), *Aggregatibacter* (2%), *Staphylococcus* (2%), and *Actinobacillus* (1%). The highest significance levels (*p* ≤ 0.002) of *Streptococcus*, *Staphylococcus*, *Actinomyces*, *Granulicatella*, *Fusobacterium*, and *Leptotrichia* were in nonsmoker samples. *Actinobacillus* was highest in smokers (*p* = 0.002). A tendency of increased abundance of *Porphyromonas*, *Neisseria*, *Haemophilus*, *Prevotella*, and *Aggregatibacter* was observed in smokers. The inner-group variance in smokers was higher than the comparison of the variance between smokers and nonsmokers.

### 3.3. Cytokines

We used a multiplex assay from Millipore to examine the expression patterns of pro- and anti-inflammatory cytokines in nonsmoker and smoker saliva samples from study participants. According to our data, IL-4, IL-2, and ACTH (*p* = 1.6 × 10^−4^, *p* = 9.07 × 10^−10^, and *p* = 5.83 × 10^−4^, respectively) were significantly higher in smoker samples. However, MDC, IL-10, IL-5, IL-7, insulin, and leptin were downmodulated compared to nonsmoker samples (*p* = 0.007, *p* = 4.28 × 10^−4^, *p* = 0.001, *p* = 0.050, *p* = 3.66 × 10^−4^, and *p* = 6.77 × 10^−4^, respectively). No significant difference was observed in the expression of TNF-α, IL-12 (p70), IFN-γ, TNF-β, IL-1 β, IL-6, IL-1ra, IL-13, IL-17, IL-7, and GM-CSF (Table 2).

### 3.4. Correlation Network

**Figure 4:**
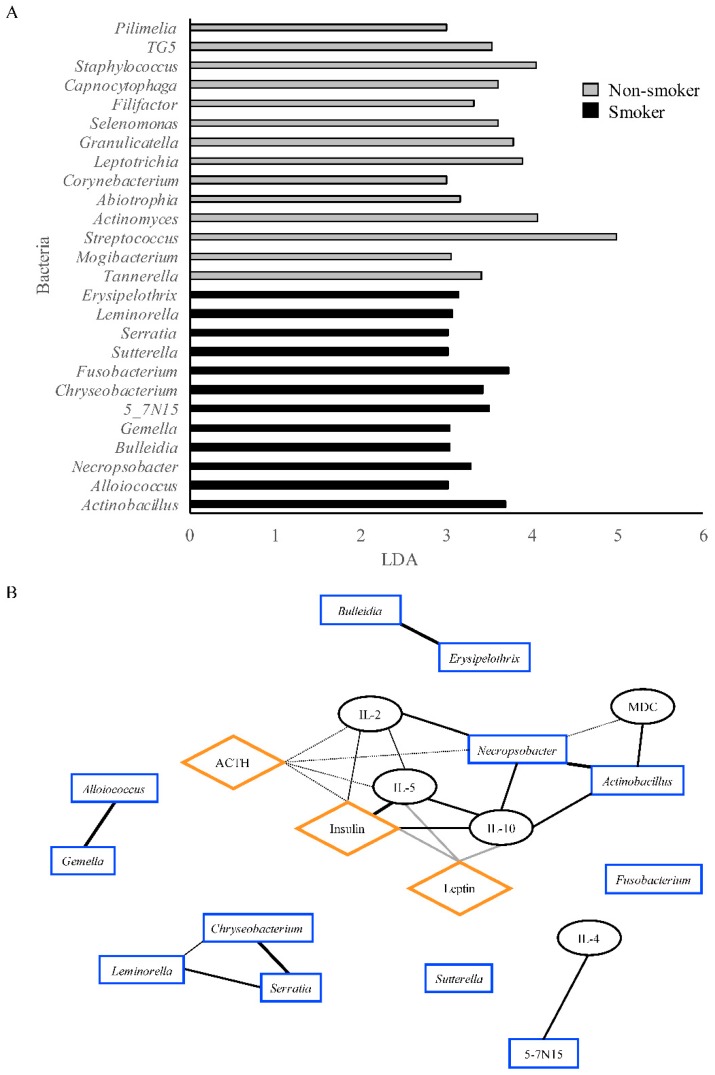
Bacteria associations and correlation network integrating hormones, cytokines, and bacteria variables enriched in the smoker group. (**A**) LEfSe-associated bacteria with nonsmoker and smoker groups, with a *p*-value < 0.05 and linear discriminant analysis (LDA) >3.0. (**B**) correlation network of bacteria associated by LEfSE in the smoker group, and statistically significant hormones and cytokines. Only significant correlations are shown as determined by qgraph. Gray lines represent negative correlations, whereas black lines represent positive correlations. The line thickness determines the strength of the correlation.

The enrichment of bacteria was done using LEfSe. The parameters used were *p* < 0.05, and the linear discriminant analysis was at least 3.0. In contrast to the genera presented in Figure 3, which were the most abundant genera, the variance of the bacteria abundance was compared between the two groups and then associated to one of the groups by linear discrimination. The LEfSe analysis resulted in 26 genera being different. Out of the 26 genera, 12 were associated with the smoker group, and 14 genera were associated with the nonsmoker group (Figure 4A). To observe the relationships between the hormones, cytokines, and bacteria identified as enriched in the smoker group, we generated a correlation network using the qgraph R package. When the smoker group-associated bacteria were combined with the cytokines and hormones in networks, we found five discrete correlation networks (Figure 4B). Interestingly, only one network contained bacteria, cytokines, and hormones, whereas the others were just one or two types of the aforementioned factors. The network that contained the most variables had two bacterial genera (*Necropsobacter* and *Actinobacillus*), four cytokines (IL-2, IL-5, IL-10, and MDC), and three hormones (insulin, leptin, and ACTH). It demonstrated that bacteria have direct correlations with other bacteria and cytokines, but indirect correlations with hormones. Thus, cytokines were the factors that were able to interact among themselves and with bacteria and hormones in a direct manner. The only negative correlations were between leptin and insulin, and between IL-10 and IL-5.

## 4. Discussion

Cigarette smoking likely modulates the microbial composition of the oral cavity. Prior studies have confirmed that tobacco smoke disrupts the homeostasis of commensal microbial composition, gingival diseases, and dental alteration [47,48]. In the present study, the use of tobacco cigarettes had a significant impact on the relative abundance of the three more prevalent bacterial phyla (Firmicutes, Proteobacteria, and Bacteroidetes) found in the saliva of samples collected in Puerto Rico. Recent analyses indicate that Firmicutes are a phylum more prevalent in saliva [49,50]. In our nonsmoker samples, Firmicutes were the most abundant phylum, while in smoker samples, Proteobacteria were the most abundant. Similarly, a significant increase in Bacteroidetes was observed in smoker samples. These findings suggest that smoking has a critical impact on oral cavity homeostasis. The positive effects of the host-microbiome symbiosis, which supports host defense functions and maintains a healthy digestive tract, may be altered by the microenvironment changes caused by the variety of organic and inorganic chemical compounds present in cigarette smoke [51,52]. Members of these phyla include some of the bacteria identified in oral diseases such as caries and periodontitis, among others. While Proteobacteria are more frequently associated with intestinal diseases, some studies indicate an association with cardiovascular disease, chronic obstructive pulmonary disease (COPD), asthma, and metabolic conditions such as diabetes [53,54].

At the genus level, the impact of cigarette smoke was observed as a lower abundance of *Streptococcus* in smoker samples. In the healthy oral cavity, species of *Streptococcus* (Firmicutes), *Actinomyces* (Actinobacteria), and *Neisseria* (Proteobacteria) are considered to be normal flora that establish a cooperative relationship with the host [55]. In contrast, some species of *Actinobacillus* (Proteobacteria), *Prevotella* (Bacteroidetes), and *Porphyromonas* (Bacteroidetes) are most often involved in the etiology of periodontitis and other oral diseases [56]. Our results showed that the frequency of these three genera in our smoker samples was significantly higher when compared to nonsmokers, suggesting an increased probability of oral infection and inflammation in the smoker participants. Additionally, recent studies have suggested that *Actinobacillus*, *Prevotella*, and *Porphyromonas* are involved in the dysregulation of cytokine networks, damage in the crevicular epithelium, leukocyte killing, the breakdown of periodontal tissues, and an increase in mucosal permeability [57,58]. The normal flora prevents oral diseases by inhibiting the adherence of pathogens onto specific surfaces, in turn preventing significant changes in the relative abundance of microbes and decreasing virulence factors [25]. Understanding that the oral cavity is one of the most important gateways of microbes into the body, we can suggest that the adverse effect of cigarette smoke on the oral microbiome will negatively impact the local and systemic compartments.

Depression symptomatology was significantly increased in the smoker group compared to the nonsmoker group. This association is consistent with previous studies, revealing that smoking and exposure to cigarette smoke can lead to the development of depression [59,60]. In addition, individuals with mental illnesses tend to smoke more than those without mental illnesses [61]. Several common pathways may explain the comorbidity of nicotine dependence and depression, including neurotransmitters such as serotonin and dopamine and the inflammatory pathways (proinflammatory cytokines). Decreased serotonin function has been related to cigarette smoking [62], and reduced serotonergic receptors to depression [63]. The nicotine in cigarettes stimulates the production of dopamine [64], which is a crucial modulator of mood and depression [65]. Additionally, both smoking and depression are associated with inflammation [66,67].

We also wanted to determine the immune response markers within the smoker group. The cytokines identified as different in the smoker group corresponded to a T-cell mediated immune response. The response was not related to one single type of T-cell, as IL-2, IL-5, and IL-10 corresponded to T_H_1, T_H_2, and T_regs_. The T_H_1 and T_H_2 responses have been reported to increase pulmonary inflammation, asthma, and allergic inflammation [68,69,70,71]. It should be noted that even though IL-5, IL-10, MDC, insulin, and leptin had lower means in the smoker group, it is interesting that they maintained the correlations that have been reported in other studies. An interpretation of this could be that there was an inflammatory response, which could then have turned into a severe dysbiosis toward metabolic syndrome, as seen in the kwashiorkor and tumor genesis scenarios [72,73]. A longitudinal study of a similar cohort could detect if the correlations of the variables indicated above may be used as early and long-term markers of metabolic syndrome in the cigarette smoking population.

When comparing the most abundant genera and the LEfSe association of the genera to the smoker and nonsmoker groups, *Necropsobacter* was identified by LEfSe as associated with the smoker group. A correlation analysis revealed that IL-2, IL-5, IL-10, MDC, insulin, leptin, ACTH, *Necropsobacter*, and *Actinobacillus* were associated within the same network. Previous work has identified IL-2, IL-5, IL-10, and MDC to be modulated by smoking [74,75,76,77,78]. Among them, IL-2 is the only cytokine that increased in the smoker group when compared to nonsmokers. A study on insulin-dependent diabetes mellitus patients revealed that there is a decreased production of IL-2 when compared to controls [79], which could indicate that the observed correlation of this cytokine with insulin in the smoker group in the present study was not a sign of insulin-dependent diabetes mellitus. In addition, the positive relationship observed between IL-2 and ACTH has been documented to play a role in depressive disorders [80,81]. Cytokine IL-5 levels were decreased in smokers, and the levels positively correlated with insulin. This observation has been detected in adipose tissue, where insulin resistance was correlated with decreased levels of IL-5 [82]. The model also positively correlated IL-5 with IL-10. The relationship between IL-5 and IL-10 has been documented as a positive one in the scenario of acute stress, in which the inflammatory profile shifts toward an anti-inflammatory state [83]. Another cytokine that had reduced expression when compared to nonsmokers was IL-10. One of the hormones that correlated with IL-10 in our model was leptin. The positive correlation of IL-10 with leptin has been documented in countering the progression of obesity and metabolic syndrome and in ameliorating inflammation [84,85,86].

Previous studies have found that smoking and smoking cessation are associated with insulin resistance [87,88,89,90], which is in accord with lower insulin levels in the smoker group. The quantification of oral insulin is supported by a previous study that measured oral insulin in smokers and demonstrated an increased risk of developing type 2 diabetes [91]. In addition to insulin resistance, tobacco induces leptin resistance [92]. A study by Cnop established the relationship between intra-abdominal fat, insulin resistance, and leptin. It demonstrated that with obesity, there is an increased risk of leptin and insulin resistance [93]. In our correlation model, leptin demonstrated a negative correlation with insulin, which has been shown to play a role in the modulation of insulin resistance in subjects undergoing a weight loss program [94]. Patients with chronic periodontitis also showed lower salivary leptin concentrations when compared to healthy volunteers [95]. Even though leptin appeared to have a lower mean in smokers when compared to nonsmokers, there was a correlation with insulin, which has been demonstrated in smokers and long-term nicotine gum users [96]. We also observed higher levels of adrenocorticotropic hormone in the smoking group. ACTH has been previously used as a marker for stress and has been positively correlated with cortisol and smoking cravings [97,98]. ACTH has also been observed to be elevated due to exposure to cigarette smoke, and could lead to metabolic syndrome [99]. Tobacco and cardiac function have been associated with changes in angiotensin-converting enzyme and ACTH levels [100].

Both *Necropsobacter* and *Actinobacillus* belong to the Pasteurellaceae family, meaning that they are closely related in terms of phylogenetic classification [101,102]. Isolation of the *Necropsobacter* genus in humans has been reported in clinical case studies related to bacteremia and abscesses [103], whereas *Actinobacillus* has been reported in the pathogenesis of periodontitis [104]. Interestingly, smoking has been linked to abscesses and periodontitis [105,106], suggesting that both *Necropsobacter* and *Actinobacillus* may have a role in the development of smoking-related abscesses.

Our study had certain limitations. First, this study had a small sample size. A larger cohort would be essential to provide stronger statistical power and to obtain robust evidence on the associations identified in our study. Second, we did not control for other possible confounders (e.g., oral health, hygiene, and physical properties of saliva). Further studies are needed to determine temporal changes in microbiome communities and immune response. Longitudinal studies will allow for identifying the time dependence of the results and the development of chronic diseases such as cancer and cardiovascular disease, among others.

## 5. Conclusions

While the study design (cross-sectional) limited the results and did not allow for the temporal association between cigarette smoking, depression, and microbiome composition, our study showed that cigarette smoking altered the expression levels of cytokines, chemokines, and hormones. It also demonstrated that the salivary microbiome differed between nonsmokers and smokers. Such differences may be a result of toxicants found in cigarette smoke, which can have an impact on host defense and metabolism and has implications for chronic diseases. The correlation analysis indicated significant correlations between oral hormones, cytokines, and bacteria, suggesting that the data obtained in this study support the use of noninvasive biomarkers in the identification of high-risk factors for the development of chronic diseases.

## Figures and Tables

**Table 1:** Summary of study subjects by smoking status.

Variable	Nonsmokers	Smokers	*p*-Value *
Individuals (*n*)	16	18	-
Age in years (mean, range)	34 (28–50)	45 (33–49)	0.109
Sex (male/female)	6/10	10/8	0.384
Race	Hispanic	Hispanic	-
Education level (percentage)			0.695
≤High school	4 (25%)	6 (33%)	
≥College	12 (75%)	12 (67%)	
Total years smoking	-	≤3 years = 2 >3 years = 15 NR ^a^ = 1	-
Cigarettes per day (number)			
Light/moderate (≤20 cigarettes)	-	9	-
Heavy (≥20 cigarettes)	-	9	-
Alcohol use (yes/no)	11/5	7/11	0.721
Assessment of depression symptoms mean ± standard deviation	6.56 ± 7.36	10.89 ± 7.04	0.020

^a^ NR: not reported; * exact *p*-values were based on Mann-Whitney U tests, with the significance level set at 0.05.

**Table 2:** Analytes in saliva samples from smokers and nonsmokers.

Analyte	Nonsmokers Mean ± Standard Deviation (pg/mL)	Smokers Mean ± Standard Deviation Mean (pg/mL)	*p*-Value *
TNF-α	28.3 ± 46.19	11.2 ± 11.33	0.646
IL-12 (p70)	8.4 ± 5.05	182.1 ± 749.43	0.281
MDC ↓	311.8 ± 399.44	114.8 ± 246	0.007
IL-10 ↓	7.7 ± 12.20	1.6 ± 0.23	4.28 × 10^−4^
IFN-γ	5.2 ± 3.50	6.9 ± 7.19	0.746
TNF-β	2.5 ± 1.12	1.8 ± 0.33	0.126
IL-1 β	443.1 ± 1358.49	214.4 ± 352.95	0.384
IL-5 ↓	2.7 ± 1.05	1.7 ± 0.12	0.001
IL-2 ↑	1.1 ± 0.02	2.4 ± 1.21	9.07 × 10^−10^
IL-6	37.1 ± 92.94	26.1 ± 71.88	0.102
IL-4 ↑	1.6 ± 1.48	15.6 ± 17.10	1.6E-4
IL-1ra	10,021 ± 2117.65	10,296 ± 4958.39	0.746
IL-13	1.9 ± 1.13	1.3 ± 0.90	0.365
IL-17	6.4 ± 10.95	8.5 ± 12.24	0.164
IL-7	11.3 ± 8.83	7.4 ± 9.90	0.050
GM-CSF	30.9 ± 53.91	22.5 ± 16.64	0.721
ACTH ↑	1.5 ± 0.80	9.2 ± 7.44	5.83 × 10^−4^
Insulin ↓	364.7 ± 87.40	27.3 ± 22.23	3.66 × 10^−4^
Leptin ↓	33.8 ± 16.03	19.3 ± 4.27	6.77 × 10^−4^

Analytes were measured using the Luminex MAGPIX (with xPONENT 4.1 program) and a cytokine magnetic bead panel (MILLIPLEX HCYTMAG-60K-PX39); (↑): significantly higher levels of analytes in the saliva samples from smokers; (↓): significantly lower levels of analytes in the saliva samples from smokers; * *p*-values were based on the Mann-Whitney-Wilcoxon test, with a significance level set at 0.05.
